# The dual regulatory effects of intestinal microorganisms and their metabolites in gouty arthritis pathogenesis: a balance between promotion and inhibition

**DOI:** 10.3389/fimmu.2025.1591369

**Published:** 2025-06-18

**Authors:** Peng Qi, Longcan Li, Jianrong Zhang, Ling Ren, Xingwen Xie

**Affiliations:** ^1^ Gansu University of Traditional Chinese Medicine, Lanzhou, China; ^2^ Affiliated Hospital of Gansu University of Traditional Chinese Medicine, Lanzhou, China

**Keywords:** gut microbiota, gut metabolites, gout, new therapeutic perspective, dual role

## Abstract

Gout is an arthritis characterized by the deposition of urate crystals, often accompanied by robust inflammatory responses. The gut microbiome profoundly influences gout pathogenesis, progression, and management by affecting uric acid metabolism, immune responses, and intestinal barrier function. Studies reveal that gut microorganisms exert a dual role in gout development. Gout patients typically exhibit increased harmful bacterial abundance and reduced beneficial species. Harmful bacteria and associated metabolites can influence systemic uric acid levels by regulating excretion and synthesis, thereby promoting gout manifestations. Conversely, beneficial bacteria interact with the host immune system to inhibit inflammation and modulate the inflammatory state of joints. Furthermore, the gut microbiome can significantly impact gout treatment efficacy through its influence on drug metabolism and absorption. Research highlighting the gut-joint-inflammation axis offers novel therapeutic strategies for gout, suggesting that future approaches may involve microbiome modulation to enhance clinical outcomes.

## 1 Introduction

Gouty arthritis (GA), a metabolic disorder arising from dysregulated purine metabolism culminating in elevated serum uric acid (UA) concentrations, constitutes a prevalent inflammatory arthritis form, characterized by tissue and organ-damaging modifications (1). Global GA prevalence is estimated at approximately 2-4%, with higher incidence in men over 40 years of age, frequently co-occurring with comorbidities, such as obesity, coronary artery disease, hypertension, and diabetes mellitus (1). GA originates from aberrant purine metabolism or diminished UA excretion, leading to monosodium urate (MSU) crystal deposition intra- and peri-articularly. Clinical manifestations encompass inflammatory symptomatology, including erythema, edema, calor, and dolor in joint soft tissues (2). Initial onset site is typically the first metatarsophalangeal joint, but it can extend to larger joints and instigate systemic acute inflammatory sequelae (3).

The human microbiome represents a critical ecosystem for sustaining health and mediating disease development (4–8). Gastrointestinal microorganisms constitute approximately 70% of the total microbial population within this ecosystem (9). The human gut microbiome is predominantly composed of *Firmicutes*, *Bacteroidetes*, *Actinobacteria*, and *Proteobacteria* phyla (10), participating in diverse physiological processes, such as nutrient metabolism, xenobiotic detoxification, immune modulation, and intestinal barrier integrity maintenance (11). Investigations indicate that the gut microbiota influences various autoimmune disease pathogenesis by modulating the host immune system (12). Upon gut microbiota dysbiosis, detrimental bacterial taxa proliferate excessively, and elaborated metabolites, such as polyphenols, vitamins, and tryptophan, may trigger GA onset (13). Furthermore, gut microorganism and metabolite alterations not only impact GA therapeutic efficacy but may also affect pharmacological agent toxic side effects. In-depth exploration of gut microorganism and GA interplay is of paramount significance for elucidating disease mechanisms, enhancing diagnostic precision, and optimizing therapeutic strategies.

## 2 The association of gut microbiome with GA

The gastrointestinal tract, the most microbial-rich human habitat, encompasses a 250–400 square meter surface area and harbors approximately 1014 microorganisms (14). Given that roughly 30% of UA is excreted via the gastrointestinal tract, the gut microbiome-GA relationship is receiving escalating attention. Gut microbiota dysbiosis is implicated in various diseases (15), encompassing hyperuricemia and GA. Gut microbes participate in purine and UA metabolism via diverse pathways: *Escherichia coli* and *Proteus* spp. secrete xanthine dehydrogenase, fostering purine conversion to UA (16, 17); *Lactobacillus* spp. inhibit intestinal purine absorption, preventing serum UA level elevations; *Pseudomonas* spp. synthesize uricase, participating in UA catabolism (18); a multitude of gut microorganisms secrete UA transporters, influencing UA excretion (19).

The gut microbiome in gout patients exhibits characteristic alterations: augmented *Prevotella*, *Clostridium*, *Bacteroides*, *Megamonas*, and *Xylanibacter* abundance, and diminished *Enterobacteriaceae*, butyrate-producing bacteria, *Coprococcus*, and *Bifidobacteria* abundance (20). Functional genomic analysis reveals increased fructose, mannose metabolism, and lipid A biosynthesis gene abundance in gout patients, and reduced UA degradation and short-chain fatty acids (SCFAs) production gene abundance (20). Diminished *Enterobacteriaceae* species abundance correlates with reduced amino acid metabolism and environmental sensing, collectively contributing to elevated serum UA and C-reactive protein concentrations (21). Liu et al., utilizing stable isotope tracing technology in a human intestinal bacterial library, identified 46 UA-degrading bacterial taxa, belonging to *Actinobacteria*, *Firmicutes*, *Clostridia*, and *Proteobacteria* phyla (22). These strains metabolize UA into xanthine or SCFAs (22). Transcriptomic analysis unveiled a highly conserved gene cluster (*ygeX*, *ygeY*, *ygeW*, *ygfK*, and *ssnA*) encoding pivotal UA degradation enzymes (23). Uricase-deficient murine model studies have confirmed that gut microbiota depletion exacerbates hyperuricemia, whereas UA-degrading bacteria colonization attenuates UA levels. Recent research indicates *Phascolarctobacterium* and *Bacteroides* enrichment in gout patients, forming a distinctive core microbiome encompassing three *Bacteroides* genera (24). Researchers have subsequently developed a 17 gout-associated bacteria diagnostic model based on these characteristics, providing novel biomarkers for gout early diagnosis and prognostic assessment (25). **Table 1** shows the correlation between intestinal microorganisms and metabolites and GA.

**Table 1:** Correlation between intestinal microbiota and metabolites and GA.

Microorganism	Effect on tumors	Mechanism
*Prevotella*,	Promote	Activate T cells, release LPS, induce metabolic endotoxemia, promote inflammation
*Bacteroides*		
*Xylan-degrading Bacteroides*		
*Escherichia coli*		Secrete xanthine oxidase, increase uric acid production from purines
*Proteus*		
*Aspartic acid*		Promote purine synthesis, increase uric acid production
*glycine*		
*LPS*		Increase intestinal permeability
*Bifidobacterium*	Inhibits	Suppress inflammation
*Faecalibacterium*		
*Blautia*		
*Ruminococcus*		
*Lactobacillus*		
*Butyrate, propionate, acetate*		
*5-HIAA*		suppress synovial cell proliferation
*kynurenic acid*		
*Lactobacillus plantarum*		Degrade uric acid or inhibit purine absorption
*L. gasseri*		
*Pseudomonas*		
*Bacillus*		
*engineered E. coli*		

## 3 Mechanisms of gut microbiota and metabolite promotion of GA development

**Figure 1:**
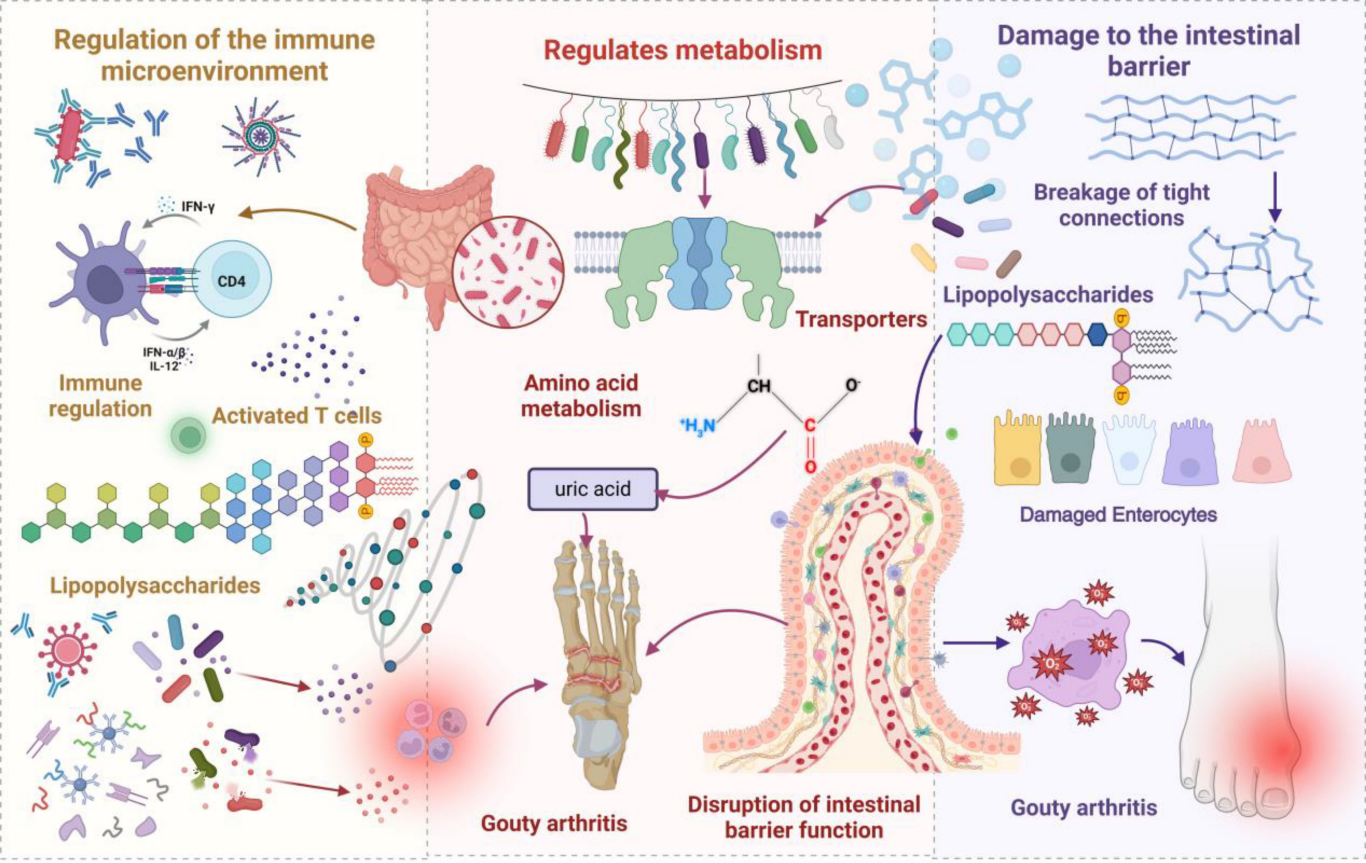
A schematic representation illustrating how gut microbiota and its metabolites contribute to the initiation and progression of gout. In the context of dysbiosis, an increase in pathogenic bacteria activates T cells, leading to the secretion of lipopolysaccharides (LPS), which triggers metabolic endotoxemia and induces systemic inflammation, thereby exacerbating gout flare-ups. Moreover, dysbiosis alters the expression of urate transporters, impeding uric acid excretion and elevating serum uric acid levels. Compromised barrier integrity facilitates the translocation of bacteria and their metabolites into the bloodstream, further amplifying inflammation and promoting the deposition of urate crystals, which increases the risk of GA.

The gut microbiota participates in GA pathogenesis via multiple regulatory mechanisms (26, 27). The abundance of specific bacteria, including *Prevotella*, *Bacteroides fragilis*, and *Xylan-degrading Bacteroides*, is significantly increased in patients with gout. This dysbiosis, marked by an expansion of pathogenic microbes, contributes to the development of GA through its impact on gut barrier integrity, metabolic regulation, and immune system function. **Figure 1** schematically illustrates gut microbiota and metabolite promotion of gout development. In innate immunity, gut microbiota dysbiosis leads to aberrant pattern recognition receptor activation, pro-inflammatory mediator expression upregulation, and anti-inflammatory mediator level downregulation, disrupting local immune homeostasis (28). At the adaptive immunity level, dysbiotic microbiota mediates autoimmune responses by modulating antigen-presenting cell function, T cell subset differentiation, and B cell activation. Microbiota dysbiosis-elicited inflammatory responses impair tight junctions between intestinal epithelial cells, augmenting intestinal permeability. Increased permeability facilitates microorganism and metabolite entry, and antigenic components, into the circulatory system, triggering systemic immune responses. These mechanisms establish a positive feedback loop: immune dysfunction exacerbates barrier functional impairment, and barrier functional damage, in turn, potentiates immunostimulatory molecule release, further amplifying immune abnormalities and leading to sustained GA progression.

### 3.1 Immunomicroenvironmental regulation

The gut microbiota orchestrates GA progression via the immune network. At the innate immunity level, gut-associated lymphoid tissue immune cells engage in signaling crosstalk with the microbiota, establishing an immune defense barrier (29). Gut microbiota dysbiosis precipitates aberrant innate immune cell activation, pro-inflammatory mediator upregulation, such as Interleukin-6, tumor necrosis factor-alpha, Interleukin-1β, Interleukin-12, and Interleukin-23, and anti-inflammatory mediator level downregulation, such as transforming growth factor-beta and Interleukin-10 (27, 30). In adaptive immune modulation, microbial antigens are recognized by dendritic cells and macrophages and presented to CD4+ T cells, inducing their differentiation (31, 32). Gut microbiota imbalance can augment intestinal permeability, leading to LPS translocation into the circulatory system, precipitating metabolic endotoxemia and inflammation (33). Elevated xanthine oxidase (XOD) activity is associated with increased serum LPS concentrations and chronic inflammation (34). Uox-KO mice exhibit significantly elevated inflammatory cytokine, LPS, and XOD activity levels (35). Compared with normouricemic mice, hyperuricemic murine models demonstrate diminished *Bifidobacterium* and *Lactobacillus* abundance in fecal samples, accompanied by elevated UA, XOD activity, and LPS levels (36), thereby accelerating GA pathogenesis and progression.

### 3.2 Metabolic Modulation

The gut microbiota and its metabolites play a crucial role in gout pathogenesis and progression by modulating UA metabolism. UA transporters in intestinal epithelial cells transport UA from the bloodstream to the intestinal lumen (37). The microbiota influences UA metabolism by modulating UA transporter expression, such as *ABCG2* and *SLC2A9* (38, 39). Purine, a precursor of uric acid, can accumulate during its metabolic processes, leading to excessive uric acid production. *Escherichia coli* and *Proteus* species are capable of secreting xanthine dehydrogenase, which catalyzes the conversion of purines to uric acid, thereby directly enhancing the host’s capacity for uric acid synthesis. This process further aggravates hyperuricemia and the development of GA. Amino acid metabolism plays a significant role in gout development. Amino acids, such as aspartic acid and glycine, participate in purine biosynthesis, augmenting UA production. Aberrant amino acid metabolism can diminish UA excretion. Furthermore, amino acid metabolites promote UA crystal deposition and exacerbate gout symptomatology by modulating immune and inflammatory responses. Gut microbiota dysbiosis may also accelerate gout onset by altering amino acid metabolism (38, 40). The gut microbiota promotes gout pathogenesis by influencing amino acid metabolism. The high-gout cluster microbiota manifests increased D/L-alanine and branched-chain amino acid metabolism, modulating UA biosynthesis (41). The abnormal accumulation of these metabolites may regulate uric acid production and levels through modulation of the purine synthesis pathway or by indirectly affecting uric acid excretion. Furthermore, amino acid metabolites may contribute to the deposition of urate crystals by modulating immune responses and inflammatory pathways, thereby aggravating the clinical manifestations of gout.

### 3.3 Intestinal barrier impairment

The intestinal barrier constitutes a multi-layered defense system, composed of the gut microbiota, mucus layer, epithelial cell monolayer, and lamina propria immune cells (42). Within the epithelial barrier, intercellular tight junction proteins play a pivotal role in maintaining barrier integrity by regulating transepithelial permeability and cellular mechanical junctions (43, 44). Increased intestinal permeability, resulting from diminished epithelial tight junction protein occludin and claudin-1 expression, exhibits a positive correlation with serum UA levels (45) (46) (47). Intestinal barrier impairment precipitates gut microbiota dysbiosis. Metabolites elaborated from gut microbiota dysbiosis, such as hydrogen sulfide, reactive oxygen species, and reactive nitrogen species, exert direct damage to intestinal epithelial cell structure and function (48), augmenting intestinal permeability, subsequently leading to bacterial translocation and increasing inflammation and gout incidence. LPS constitutes the principal Gram-negative bacteria cell wall component. Gut micro-ecological imbalance can markedly inhibit Gram-negative bacteria physiological activity, leading to increased LPS elaboration. Excessive LPS can induce pro-inflammatory cytokine production and augment intestinal barrier permeability, precipitating metabolic endotoxemia (49). In gout pathogenesis, gut microbiota dysbiosis promotes LPS translocation into the bloodstream by impairing intestinal barrier function, leading to metabolic endotoxemia. This process not only escalates the systemic inflammatory burden but can also exacerbate UA accumulation and gout onset by affecting renal function (50).

## 4 Gut microbiota and metabolite mechanisms inhibiting GA development

**Figure 2:**
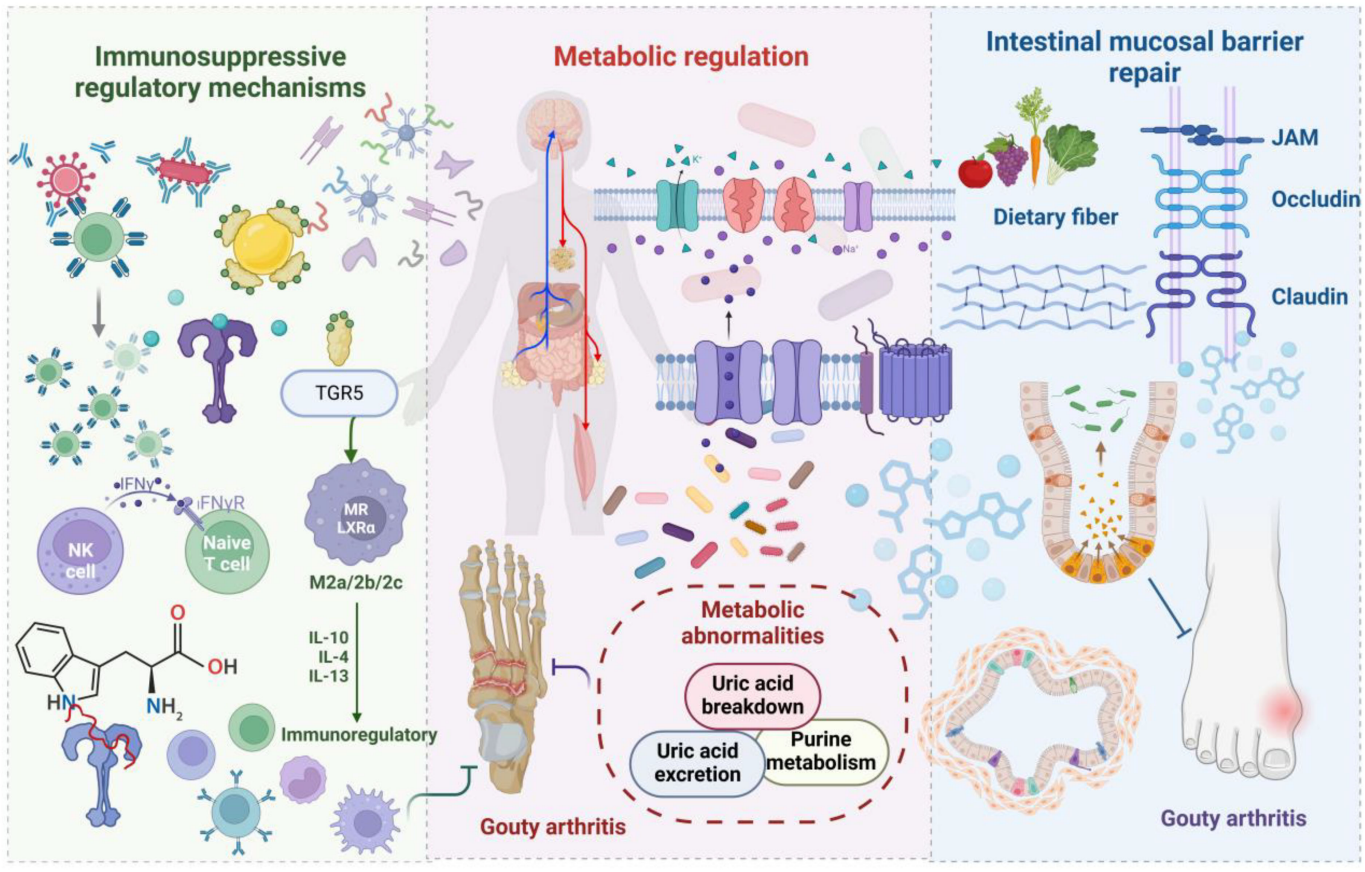
A schematic representation illustrating how gut microbiota and its metabolites suppress the onset and progression of gout. Beneficial bacteria and their metabolites mitigate inflammation through immune modulation mediated by TGR5, while uricase reduces systemic uric acid accumulation. Nutritional factors, such as dietary fiber, enhance microbiota composition, preserve gut barrier integrity, and decrease the permeability to harmful substances. These mechanisms work in concert to inhibit the development and recurrence of GA.

Gut microbiota dysbiosis exerts a dual regulatory role in GA pathological progression. Beneficial bacterial communities play a protective role by modulating immune responses and maintaining intestinal barrier integrity. Investigations have revealed significantly reduced *Faecalibacterium* and *Bifidobacterium catenulatum* abundance in gout patients (21). Metagenomic analysis has corroborated decreased *Enterobacteriaceae* bacteria and butyrate-producing bacteria numbers within gout patient intestines (38). In a hyperuricemic nephropathy rat model, beneficial bacterial taxa, such as SCFA-producing *Blautia* and *Ruminococcus* genera, were markedly diminished. **Figure 2** schematically illustrates gut microbiota and metabolite inhibition of gout development.

### 4.1 Immunosuppressive regulatory mechanisms

Gut microbiota and their metabolites inhibit GA pathogenesis and progression by modulating immune cell function and inflammatory cytokine expression. The NLR family pyrin domain Containing 3 (NLRP3) inflammasome, functioning as a metabolic stress sensor, participates in gout development (51–53). *Lactobacillus* spp. inhibit NLRP3 activation by restoring mitochondrial membrane potential, and *Bifidobacterium longum* and *Bacteroides fragilis* suppress NLRP3 activation via inflammatory signaling pathway inhibition (54–56). *Lactobacillus casei* elicits anti-inflammatory responses by interleukin-10 expression upregulation and Interleukin-6 and tumor necrosis factor-α level downregulation. *Lactobacillus rhamnosus* mediates immunosuppression by modulating Interleukin-1 expression. *Phascolarctobacterium praecalvibacter* reduces pro-inflammatory mediator concentrations, Interleukin-17, Interleukin-1β, and tumor necrosis factor-α, while concurrently promoting symbiotic microbiota proliferation, such as *Akkermansia* and *Bifidobacteria* (57, 58). Microbial metabolites play a crucial role in immunomodulation. Butyrate, a SCFA, promotes follicular regulatory T cell differentiation (59). *Parabacteroides distasonis*-produced bile acid metabolites induce macrophage M2 polarization and inhibit Th17 cell differentiation via TGR5 receptors (60). The tryptophan metabolic network plays a salient role in GA immunomodulation: 5-hydroxyindoleacetic acid induces regulatory T cell differentiation via aromatic hydrocarbon receptors (61); kynurequinolic acid inhibits synovial cell proliferation (62); 3-hydroxyanthranilic acid suppresses inflammatory responses by blocking the NF-κB signaling pathway (63). *Blautia*- and *Roseburia*-elaborated butyrate attenuates GA flares by inhibiting MSU crystal-induced Interleukin-1β, Interleukin-6, and Interleukin-8 production.

4.2 Metabolic regulation

Gut microorganisms regulate UA metabolism via multiple enzymatic systems, and certain beneficial bacterial taxa exhibit UA oxidase and xanthine dehydrogenase inhibitory activities. *Lactobacillus* and *Pseudomonas* spp. promote UA catabolism and excretion by producing SCFAs (64). The *Lactobacillus* genus catabolizes inosine and guanosine to inhibit UA biosynthesis (65–67). *Lactobacillus* OL-5, *Lactobacillus plantarum* Mut-7, and *Lactobacillus plantarum* Dad-13 exhibit elevated uricase activity and can degrade UA via uricase, thereby reducing *in vivo* UA accumulation (37). *Lactobacillus gallinarum* reduces intestinal purine concentrations (68). *Lactobacillus gasseri* diminishes purine absorption (18). Propionate and butyrate promote UA excretion by supplying adenosine triphosphate (69, 70). *Bacillus*, *Proteus mirabilis*, *Escherichia coli*, and various other microorganisms can degrade UA via uricase, thereby reducing *in vivo* UA accumulation (71) (72), consequently inhibiting GA development.

### 4.3 Intestinal mucosal barrier restoration

Aberrant intestinal barrier function is intimately associated with autoimmune disorders. Nutritional factors participate in barrier repair by modulating microbial composition and metabolic pathways. Dietary fiber maintains barrier integrity by reducing serum Zonulin and calprotectin levels (73) (74). Vitamin D modulates epithelial cell tight junctions and apoptosis (75). Vitamin E promotes butyrate-producing bacteria proliferation (76). Glutamine and tryptophan deficiency result in barrier function impairment (77, 78). Plant polyphenols confer barrier function protection by enhancing transepithelial electrical resistance and ZO-1 and claudin-1 expression upregulation (79). Butyrate repairs intestinal epithelial cells and stabilizes the epithelial mucosal barrier (72). Acetate provides energy substrates for intestinal epithelial cells and promotes UA transport (72). *Bifidobacteria* ameliorate mucosal barrier function by inhibiting detrimental strain proliferation (80), and SCFAs mediate mucosal barrier repair (81), thereby inhibiting GA progression.

## 5 Translational gut microbiome applications in GA diagnosis and therapeutics

Gut microbiota composition and diversity alterations participate in GA pathogenesis and progression via immune system modulation (82). Gut microbiome-based intervention strategies have demonstrated preliminary therapeutic efficacy in GA clinical investigations. Animal studies have substantiated that probiotic supplementation and intestinal barrier stability maintenance, among other measures, possess potential therapeutic value for GA (83), suggesting that precision microbiome composition modulation may facilitate novel diagnostic biomarker and personalized therapeutic regimen development.

### 5.1 Diagnostic biomarkers

Predicated on the gut microbiome-gout development correlation, gout-specific microbiota may serve as potential diagnostic biomarkers. Lin et al., based on bacterial genera exhibiting significant disparities between healthy individuals and gout patients, constructed a classification model with an area under the receiver operating characteristic curve reaching 0.973 (84). Another cohort study established a 17 gout-associated bacteria diagnostic model, achieving 88.9% accuracy (20). Chu et al., via metagenomic analysis, identified three genes significantly enriched in the gout cohort, with development and validation cohort area under the receiver operating characteristic curve of 0.91 and 0.80, respectively (21). Gout-characteristic gut microbiota imbalance may serve as a non-invasive diagnostic tool for gout and asymptomatic hyperuricemia, providing novel prevention and intervention targets. The significance of gut microbiota characteristics extends beyond current diagnostic applications, with dynamic changes also being explored as potential prognostic indicators for disease progression and treatment response. Monitoring shifts in the gut microbiota structure and function in gout patients undergoing urate-lowering therapy or probiotic interventions can provide valuable insights into treatment efficacy, while also offering the potential to predict disease progression and relapse risk. To more deeply investigate the complex interplay between gut microbiota and the immune-pathological processes of gout, advanced technologies are increasingly being applied to identify relevant biomarkers (85). Spatial transcriptomics and spatial proteomics offer the ability to perform high-resolution analyses of local microenvironments—such as synovial and renal tissues—while preserving spatial context, allowing for precise mapping of microbial components, immune cell populations, inflammatory mediators, and urate crystal distribution, along with their interactions. By correlating this localized microenvironmental data with global gut microbiota profiles obtained via high-throughput sequencing, we can uncover how distal gut microbial signals impact local joint inflammation or renal damage, thereby identifying spatial biomarkers more tightly associated with gout onset, progression, and prognosis (86). These integrative approaches offer novel insights into how gut microbiota modulate host immunity and regulate local microenvironments in the context of gout, paving the way for the discovery of mechanistically targeted biomarkers with potential clinical diagnostic, prognostic, and predictive applications.

### 5.2 Gut Microbiota and Metabolite-Based GA Therapies

Non-steroidal anti-inflammatory drugs, glucocorticoids, and colchicine constitute first-line pharmacological agents for acute gout management (87). XOD inhibitors and uricosuric agents serve as first- and second-line choices, respectively, for UA-lowering therapy (88). Gut microbiota dysbiosis can impact therapeutic outcomes (89): GA patient gut microbiota composition undergoes post-treatment alterations, promoting SCFA and acetate production (90).

#### 5.2.1 Post-traditional pharmacotherapy gut microecology alterations

Non-steroidal anti-inflammatory drugs can disrupt gut microbiota equilibrium, fostering Gram-negative bacteria proliferation and inhibiting Gram-positive bacteria growth. Microbiota dysbiosis activates inflammatory responses via the TLR4 pathway and augments intestinal permeability (91, 92). Colchicine blocks microtubule protein polymerization, precluding inflammasome activation (93). However, colchicine demonstrably impacts gastrointestinal architecture, altering gut microbiota diversity and composition, resulting in pro-inflammatory mediator downregulation and intestinal barrier impairment (94). Allopurinol therapy can augment *Bifidobacteria* abundance and reduce anaerobic bacteria numbers (89). *Bilophila* genera, as the sole reducing genus, can induce systemic inflammation (95). Benzbromarone reduces UA concentrations by blocking URAT-1 (96), while concurrently modifying gut microbiota composition, increasing *Bifidobacteria* and reducing butyrate-producing bacteria (89). Febuxostat inhibits XOD activity and can partially restore gut microbiota diversity in gout patients (66). Functional analysis reveals enhanced purine metabolism potential of gut microorganisms in post-treatment patients (84), and micro-inflammation suppression (97).

#### 5.2.2 Microbiome-mediated therapeutic efficacy modulation mechanisms

**Figure 3:**
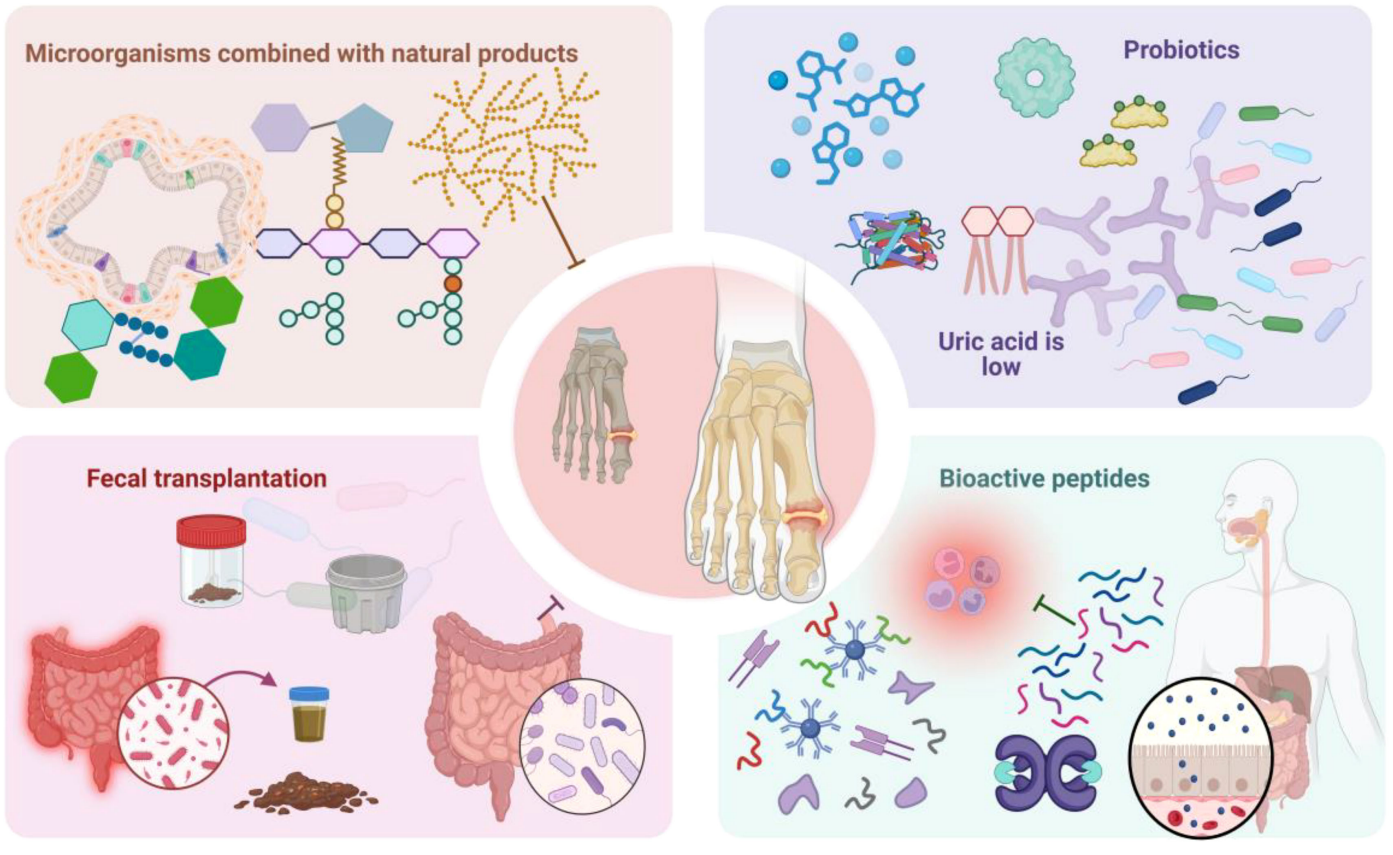
Schematically illustrates microbiome-mediated therapeutic approaches. Microbiome-mediated therapies encompass probiotic modulation, microbial combination with natural products, FMT, bioactive peptides, and other modalities. Among these, probiotics regulate gut microbiota structure and function to ameliorate gout symptomatology. Microbial combination with natural products, FMT, bioactive peptides, and other therapeutic interventions can effectively reduce serum UA levels, attenuate inflammatory responses, and inhibit GA pathogenesis and progression, providing novel paradigms for GA treatment.

Novel therapeutic modalities, such as natural products, probiotics, and fecal microbiota transplantation (FMT), regulate gout pathogenesis and progression via multiple mechanisms (98–100). **Figure 3** schematically illustrates microbiome-mediated therapeutic approaches. These modalities inhibit purine metabolism and inflammasome activation, regulate transporter protein expression, and maintain intestinal barrier integrity. Concurrently, they can augment SCFA-producing bacteria abundance, thereby inhibiting XOD activity and achieving UA-lowering effects (101). These interventions provide novel paradigms and potential targets for gout therapy.

(1) Probiotics

Probiotics, such as *Bifidobacteria* and *Lactobacillus* genera, ameliorate gout symptomatology by modulating gut microbiota structure and function (102). Novel probiotics, including *Faecalibacterium prausnitzii*, *Akkermansia muciniphila*, and *Clostridium* spp., also demonstrate therapeutic potential (103). *Lactobacillus fermentum* JL-3 can rectify gut microbiota dysbiosis associated with hyperuricemia (99). Probiotic *strain DM9218* diminishes serum UA concentrations and hepatic XOD activity (104). Uricase-elaborating bacteria-containing probiotics can improve hyperuricemia and confer renal function protection (105). Prebiotics, functioning as selective substrates for host microorganisms (106), can augment *Lactobacillus* and *Bifidobacteria* abundance and foster butyrate and propionate production (107), providing novel therapeutic avenues for gout. In a diet-induced hyperuricemic murine model, *EH-JAP* and *EH-LEU* improved hyperuricemia and GA by inhibiting UA biosynthesis and promoting UA excretion. Concurrently, these two therapeutic regimens improved gut microbiota functionality by increasing beneficial *Lactobacillus* and SCFA-producing bacteria abundance and reducing opportunistic pathogen numbers (37).

(2)Microbial combination with natural products

Microbial combination with natural products can effectively diminish serum UA levels and inhibit GA pathogenesis and progression. Luteolin attenuates serum UA concentrations in GA mice by modulating gut microbiota composition, reversing *Bacteroidetes* and *Firmicutes* phyla dysbiosis. Investigations suggest that luteolin also ameliorates renal function in GA-induced chronic kidney disease mice by modulating gut microbiota-mediated tryptophan metabolism (108). Metabolomic analysis indicates that nuciferine can inhibit hyperuricemia development by modulating impaired metabolic pathways and gut microbiota composition (109). *Ulva pertusa* polysaccharide, the most prevalent nuciferine family green algae extract, not only reduces serum UA levels but also significantly enhances gut microbiota diversity, particularly increasing *Alistipes* and *Parasutterella* genera abundance. Correlation analysis reveals *Parasutterella* content exhibiting a negative correlation with UA levels (110). Therefore, microbial combination with natural products offers novel gout therapy strategies.

(3) Fecal microbiota transplantation

FMT can reduce serum UA concentrations in gout patients and diminish acute gout flare frequency and duration (37). Simultaneously, FMT can reduce diamine oxidase and endotoxin levels and ameliorate impaired intestinal barrier function (100). Animal experiments further substantiate FMT potential value in hyperuricemia therapy. Furthermore, FMT effectively alleviates hyperuricemia in mice by selectively modulating AJOP-related metabolic pathways, suggesting AJOP protective effect partially contingent upon microbiota modulation (111). Goose essence-treated mice FMT also demonstrates significant anti-hyperuricemic effects, with mechanisms encompassing gut microbiota equilibrium restoration, intestinal epithelial barrier repair, and SCFA production promotion (112). Although FMT long-term human health effects necessitate further clinical validation, it offers a novel gout treatment option and possesses significant clinical application prospects.

(4)Bioactive peptides

Bioactive peptides constitute a key research domain in food, health products, and specialized medical foods. These polypeptides are not only elaborated from intestinal microbial proteases acting on dietary proteins but also significantly modulate gut microbiota structure, consequently impacting host health. Tuna oligopeptides ameliorate hyperuricemia and GA by reprogramming UA metabolic pathways, inhibiting NLRP3 inflammasome and TLR4/myeloid differentiation primary response 88/nuclear factor-kappa beta signaling pathway activation, and p65-nuclear factor-kappa beta phosphorylation. Concurrently, tuna oligopeptides repair the intestinal epithelial barrier, rectify gut microbiota dysbiosis, and promote SCFA production (113). Investigations reveal that hexapeptides *GPAGPR* and *GPSGRP*, as potential microbiota modulators, reduce serum UA levels by inhibiting UA biosynthesis and reabsorption, and attenuate renal inflammation by inhibiting NLRP3 inflammasome activation. These two peptides also diminish gut microbiota richness and diversity, altering phylum and genus level microbiota composition (114). Sea cucumber oligopeptides significantly alleviate hyperuricemia, with action mechanisms encompassing UA metabolism modulation, NLRP3 inflammasome and nuclear factor-kappa beta-related signaling pathway activation inhibition, and m6A methylation level restoration (111).

## 6 Conclusion

GA is a complex autoimmune disorder with multifactorial pathogenesis, wherein gut micro-ecological imbalance plays a pivotal role in disease progression. GA patients exhibit characteristic alterations, including detrimental bacteria increase and beneficial bacteria decrease. Gut microbiota and metabolites participate in GA regulation through multiple pathways: detrimental bacteria promote GA pathogenesis and progression by fostering inflammation and disrupting the intestinal barrier, whereas beneficial bacteria inhibit GA pathogenesis and progression via inflammation suppression and intestinal barrier repair. Gut microbiota and metabolites possess potential applicability in GA diagnosis and therapy. Microbiota composition analysis can predict GA susceptibility and has emerged as an efficacious methodology for morbidity prediction and control. Furthermore, gut microbiota and enzymatic products can directly or indirectly influence pharmaceutical agent bioavailability, clinical efficacy, and toxicity, while therapeutic drugs and active compounds can also modulate immune cell function by normalizing gut microbiota composition.

While therapeutic strategies targeting the gut microbiome have demonstrated potential for modulating the immune system, current research faces several limitations. The molecular mechanisms by which specific microbial communities or metabolites regulate host immune responses and drive the pathogenesis of GA remain elusive. Precise mechanisms linking gut microbiota dysbiosis and impaired intestinal barrier function to the induction of systemic inflammatory responses also warrant further investigation. Moreover, applying microbiota modulation strategies for GA therapy is challenging, primarily due to significant inter-individual variations in gut microbial composition, immune responses, and therapeutic efficacy, which can substantially impact intervention outcomes. However, leveraging continuous advancements in genomics, microbiology, and immunology, coupled with multidisciplinary integrated analyses, the development of personalized microbiome-based interventions holds promise for enhancing the precision and effectiveness of GA therapy. Future efforts must prioritize exploring the intricate microbiota-immune interactions to facilitate the development of truly personalized therapies. Rigorous systematic clinical studies and robust experimental data are essential to fully realize the potential of microbiota modulation as a novel therapeutic strategy for GA.
